# Pathway expression analysis

**DOI:** 10.1038/s41598-022-26381-x

**Published:** 2022-12-17

**Authors:** Nathan Mankovich, Eric Kehoe, Amy Peterson, Michael Kirby

**Affiliations:** Colorado State University, Mathematics, Fort Collins, 80523 USA

**Keywords:** Bioinformatics, Microarray analysis

## Abstract

This paper introduces a pathway expression framework as an approach for constructing derived biomarkers. The pathway expression framework incorporates the biological connections of genes leading to a biologically relevant model. Using this framework, we distinguish between shedding subjects post-infection and all subjects pre-infection in human blood transcriptomic samples challenged with various respiratory viruses: H1N1, H3N2, HRV (Human Rhinoviruses), and RSV (Respiratory Syncytial Virus). Additionally, pathway expression data is used for selecting discriminatory pathways from these experiments. The classification results and selected pathways are benchmarked against standard gene expression based classification and pathway ranking methodologies. We find that using the pathway expression data along with selected pathways, which have minimal overlap with high ranking pathways found by traditional methods, improves classification rates across experiments.

## Introduction

Influenza and other viruses linked to respiratory illnesses in humans have gained relevance due to the recent COVID-19 pandemic caused by the severe acute respiratory syndrome coronavirus 2 (SARS-CoV-2). Accurate detection of these types of viruses is necessary to isolate infected individuals and consequently slow the spread throughout the population. Moreover, some infected individuals can be shedding the virus without showing symptoms. It is advantageous to have methods of detecting shedding that are robust to symptoms to avoid asymptomatic super spreader scenarios^1^.

Analyses of respiratory illnesses aid in understanding the mechanisms of shedding and in developing methodologies that succeed across multiple infectious diseases. Understanding the imprint of viral shedding on human gene expression may uncover latent effects which are beyond disease symptoms. In this paper we will run our experiments on a human microarray data set with multiple respiratory viruses and studies, the GSE73072 data set^2^. Previous work on these respiratory virus data ran various machine learning (ML) models, e.g., neural networks, support vector machines (SVM), centroid encoders (CE), and spectral gene network analysis, to identify discriminatory biomarkers within early shedders challenged with influenza and subsequently classify those subjects^3–6^.

Rather than just select significant genes, a biological pathway analysis uses biological relationships between genes, usually by grouping related genes, to build a biologically informed model^7^. Given the numerous definitions of pathway membership and number of ways to relate genes within a pathway, many of these pathway analyses require an a priori set of “important” genes (like those found using ML on gene expression data^3^) to determine significant pathways^8,9^. Standard tools that rely on an a priori gene set include: over representation analysis (ORA)^10^, gene set enrichment analysis (GSEA)^11–14^, Centrality-based pathway enrichment (CePa)^15^ and more^16^. ORA determines the statistical significance of the overlap between an a priori set of genes and a given pathway. GSEA improves upon ORA by accounting for the expression levels of the genes. CePa is a further improvement on the ORA method which uses the biological connections between genes in a pathway. Specifically, it uses the centrality of genes in the network generated by a pathway as part of its ranking. Beyond simply ranking pathways by statistical significance, Maglietta et al. offer a method that ranks functional groups of genes, specifically genes associated with the same GO term^17^, to predict a phenotype^16^. All of these pathway methods rely on a pathway database which is used to determine pathway membership. There are a plethora of pathway databases including KEGG^18^, MetaCyc^19^, Reactome^10^, Wikipathways^20^, BioCarta^21^, InnateDB^22^ and many more. Most of these databases contain essentially the same information regarding pathway membership. We use the Reactome database, along with their built in tool for ORA in order to produce pathway rankings to compare against our novel pathway expression analysis rankings.

In this paper, we formalize a method called pathway expression analysis which transforms gene expression data into *pathway* expression data. This allows for the determination of pathway significance and subject classification using pathways expression levels rather than genes expression levels. This is not the first time such a translation has been considered. Some of the following works use GO terms or other biologically known collections of genes rather than pathways. For the sake of simplicity, we will use the term pathway loosely in this paragraph to refer to a biologically known collection of genes. Between the early 2000’s and 2012, various authors have explored classification and pathway ranking using statistical techniques. Pathway expressions, often referred to as pathway activities, have been calculated using means and medians^23^, metagenes (aka. eigengenes)^24^, *t*-scores^25^, log-likelihood ratios (LLRs)^26^, and FAIME (Functional Analysis of Individual Microarray Expression)^27^. Some of these methods do not use all the genes in the pathway to calculate pathway expression. For example Guo et al. only calculate the average expression level of the differentially expressed genes in a pathway. Pathway selection in these papers is not always done using pathway expression levels. Su et al.^26^ use gene expression levels to select pathways. On the other hand, some works do indeed use their pathway expression data to determine significant pathways and to classify between phenotype. Lee et al. determine significant pathways for classification using the AUC (area under the ROC curve) on a validation data set^25^. Classification and regression of changes in phenotype with pathway expression data has been done using simple models like decision trees^23^, linear discriminant analysis^26^, logistic regression^25,26^, clustering^24,27^ and other statistical models^27^.

The last known work in this field is FAIME in 2012^27^. Within the last 10 years, the field of machine learning in biology has boomed in popularity and there’s a need for *formalization* of these previous pathway expression methods. Our work fills this gap by defining pathway expression data as the result of a simple linear transformation of gene expression data. We offer two forms of pathway expression data: Linear Pathway Expression (LPE) and Centrality Pathway Expression (CPE). Methods like those presented by Guo et al. fall easily into the pathway expression framework as a special case of LPE^23^. CPE, on the other hand, is a novel method for pathway expression that ranks genes based on networks of known and inferred gene interactions rather than statistically inferring a gene ranking using differences between phenotype like the FAIME method^27^. Unlike other previous pathway expression methods (e.g., FAIME), CPE and LPE can be thought of as ’unsupervised’ pathway expression methods since they do not require phenotype labels for their computation. Due to their simplicity, CPE and LPE use less computational resources than most previous pathway expression methods since our methodology involves a linear transformation rather than more complicated non-linear mappings. Rather than use one of the classifiers from the previous works we choose to use Sparse Support Vector Machines (SSVMs) for our classifier. This sparse classification scheme allows us to preform our pathway selection and classification using pathway expression data in one step, without any parameter tuning.

We use our pathway expression methods (LPE and CPE with SSVM) to select pathways which discriminate between uninfected subjects (controls) and eventual shedders infected with various respiratory viruses: H1N1, H3N2, HRV (Human Rhinoviruses), RSV (Respiratory Syncytial Virus), in the early stages of infection using the GSE73072 data set. O’Hara et al. found that gene expression data from some pathways like B Cell Maturation and Activation + Cell Adhesion Molecules were used to produce 100% classification accuracy for certain experiments with this data set but analyses with pathway expression has never been done^6^. This work compliments the early detection analysis done by Aminian et al.^3^ by utilizing “pathways expression” with a simple feature selector and classifier in place of gene expression with optimal feature selection and classification techniques. By using pathway expression as an alternative to gene expression, we extract pathways as features using SSVMs and subsequently rank pathways by their weight in the SSVM model. We do this by extracting the top pathways for each pathway expression method on training data, restricting the test pathway expression data to those top pathways, and finally classifying controls vs. shedders (from 4 evenly spaced time bins within 32 hours after infection) using the test pathway expression data using SVM. We also benchmark these selected pathways against two known pathway ranking methodologies ORA and CePa. As an aside, our implementation of CePa in Python, to the best of our knowledge, is one of the first Python implementations of such a workflow.

In our experiments on the GSE73072 dataset we find that pathway expression methods generally produce higher classification rates than gene expression methods with the same type of SSVM feature selector and classifier. We also find that using CPE, which adds gene network information, increases classification rates over simple LPE. Classification rates with CPE and LPE are found to be robust LIMMA normalization of the gene expression data and the pathways selected by these methods prove to be appropriately robust across training dataset partitions. The pathways which are selected using CPE and LPE methods tend to be distinct from the pathways selected using CePa and ORA. Hence, pathway expression is a useful method for discovering biological pathways which are not traditionally associated with a disease of interest. The code for LPE and CPE is implemented in Python and available at https://github.com/nmank/PathwayAnalysis. This work is meant to revive work in pathway expression analysis by adding a simple framework for generating pathway expression data, introducing a new method for pathway expression, CPE, which leverages gene networks to produce pathway expression data and finally implementing a simple sparse classifier to select pathways and discriminates between phenotype at the same time.

## Results

In this section we provide a number of results using pathway expression on GSE73072. Specifically, we investigate two types of pathway expression: Centrality Pathway Expression (CPE) and Linear Pathway Expression (LPE) and compare these methods to Gene Expression (GE) methods. We provide a visualization of pathway expression data in Supplementary Information. This section is broken down into Classification Results, Comparing Pathway Selection Methodologies and Top CPE Pathways. In Classification Results we provide classification statistics including Balanced Success Rates (BSRs) for experiments using the features selected on pathway expression on test studies. We also pose an argument for the best CPE method in Classification Results. In Comparing Pathway Selection Methodologies, we compare the pathways selected using pathway expression to those selected using CePa and ORA. Lastly, in Top CPE Pathways, we list the top pathways found using the best CPE method.

### Classification results

In our classification experiments we draw samples from the GSE73072 data set and distinguish between controls and shedders within 32 hours after infection. For our experiments, we separate these data into 4 evenly sized time bins. Controls are all subjects at times before infection. We perform 3 different data set splits. For two of these splits, we find features using data from 4 studies and on the remaining 2 or 3 studies. For the third split, we find features using data from 6 studies then test on 1 study. The details of the data sets used for these experiments are in Supplementary Information.

We use these classification experiments to compare LPE and CPE methods to each other and benchmark these pathway expression methods against GE methods. Classification results on the test studies from the 4 to 2, 4 to 3 and 6 to 1 experiments with LIMMA batch correction by subject ID are reported Table 1. A comparison between test classification BSRs between uncorrected and LIMMA corrected data which indicates that pathway expression classification BSR is more robust to LIMMA correction than gene expression using our pipelines is in the Supplementary Information. Using pathway expression, LPE or CPE, we produce the same or higher classification statistics on the test data than GE for more than 81% of all the test statistics, experiments and time bins.

**Table 1:** Classification statistics by method, experiment and time bin on the test data sets. Pathway expression methods generally produce a higher BSR, precision, recall and accuracy over gene expression methods with the SVM feature selection technique.

Time bin	Experiment	Method	BSR	Precision	Recall	Accuracy
1–8	4–2	GE	0.6	0.71	0.71	0.64
1−8	4–2	LPE	0.72	0.79	0.81	0.73
1–8	4–2	CPE	**0.8**	**0.85**	**0.86**	**0.8**
9–16	4–2	GE	0.74	0.81	0.76	0.72
9–16	4–2	LPE	**0.82**	**0.87**	**0.85**	**0.8**
9–16	4–2	CPE	0.74	0.8	0.8	0.73
17−24	4–2	GE	0.67	0.74	0.76	0.69
17–24	4–2	LPE	0.7	0.77	0.77	0.7
17–24	4–2	CPE	**0.77**	**0.83**	**0.82**	**0.77**
25–32	4–2	GE	**0.96**	**0.98**	**0.99**	**0.98**
25–32	4–2	LPE	0.92	0.97	0.96	0.94
25–32	4–2	CPE	0.9	0.96	0.94	0.92
1–8	4–3	GE	0.61	0.74	0.71	0.65
1–8	4–3	LPE	**0.73**	**0.83**	0.8	0.76
1–8	4–3	CPE	**0.73**	0.82	**0.84**	**0.77**
9–16	4–3	GE	0.74	0.83	0.78	0.74
9–16	4–3	LPE	0.79	0.86	**0.85**	0.78
9–16	4–3	CPE	**0.81**	**0.88**	0.84	**0.81**
17–24	4–3	GE	0.59	0.72	0.65	0.6
17–24	4–3	LPE	0.69	0.79	0.75	0.69
17–24	4–3	CPE	**0.77**	**0.85**	**0.83**	**0.78**
25–32	4–3	GE	0.8	0.91	0.87	0.84
25–32	4–3	LPE	0.8	0.91	0.87	0.84
25–32	4–3	CPE	**0.83**	**0.92**	**0.92**	**0.88**
1–8	6–1	GE	0.62	0.8	**0.97**	0.81
1–8	6–1	LPE	0.8	0.89	**0.97**	0.89
1–8	6–1	CPE	**0.89**	**0.94**	**0.97**	**0.93**
9–16	6–1	GE	**0.91**	**0.94**	**1.0**	**0.96**
9–16	6–1	LPE	0.89	**0.94**	0.97	0.93
9–16	6–1	CPE	0.82	0.91	0.91	0.86
17–24	6–1	GE	0.77	0.87	**1.0**	**0.9**
17–24	6–1	LPE	0.74	0.86	0.94	0.85
17–24	6–1	CPE	**0.8**	**0.89**	0.97	**0.9**
25–32	6–1	GE	0.76	0.87	0.97	0.87
25–32	6–1	LPE	0.68	0.84	0.91	0.81
25–32	6–1	CPE	**0.91**	**0.94**	**1.0**	**0.96**

CPE is computed using pre-computed edges with PageRank centrality. The highest statistic for each time bin is bold face. All experiments apply LIMMA using subject identifier to the data. Standard deviation is not available for these statistics due to the design of our LOSO experiments. See the Methods section for details.

CPE requires two parameters to be set: a pathway gene network edge type and a centrality measure. We test CPE by using either correlation edges or pre-computed (from Reactome) edges, which may be directed or undirected. For centrality measures, we use either PageRank or out-degree centrality. Supplementary Information is a table of the CPE configuration which produces the highest test BSR. Using this table, we observe that pre-computed edges with PageRank centrality is the most common method across all experiments and time bins to produce the highest BSR.

**Figure 1:**
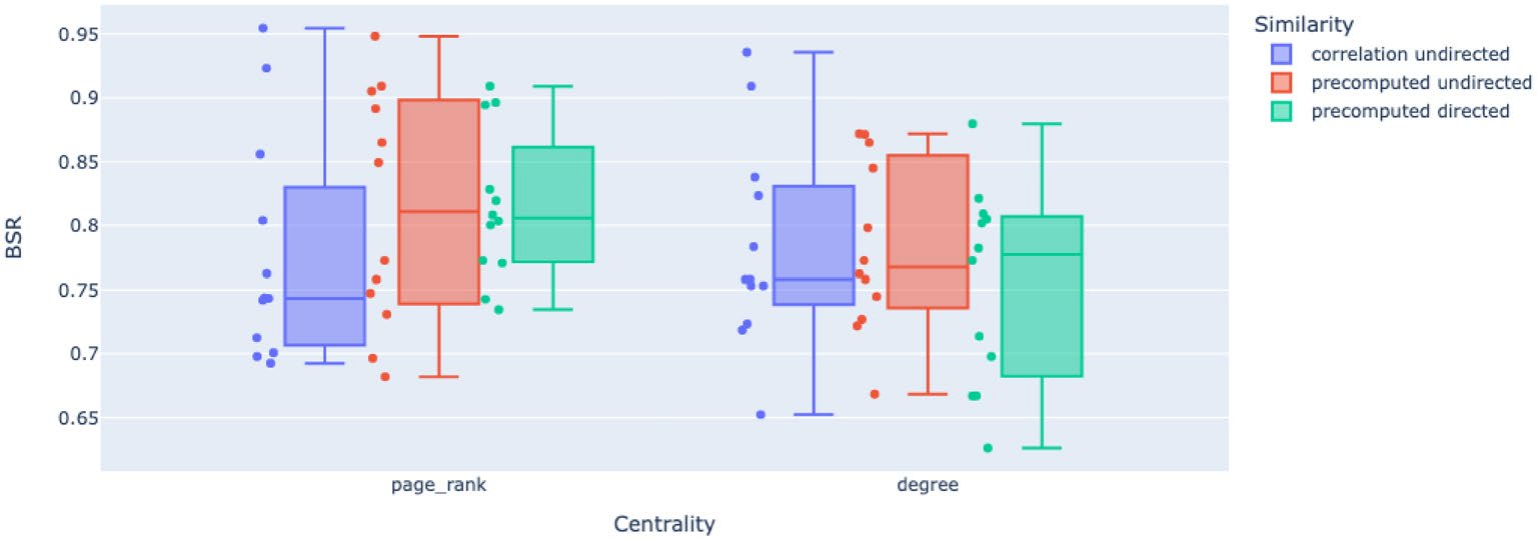
The distribution of BSR across experiments for each CPE configuration. Notice pre-computed, directed edges with PageRank centrality has the one of the highest BSRs while maintaining the lowest variance. All experiments apply LIMMA using subject identifier to the data.

Finding the “best” CPE configurations by using the configurations which produce the maximum test BSR is not the best way to select the “best” CPE configuration since it doesn’t take into account the performance of each CPE configuration across all experiments. So, in order to choose the best CPE edge type and centrality ranking, we look at the distributions of test BSRs for each edge type and centrality ranking across experiments. The box plot from this investigation is in Fig. 1. We believe that the best CPE configuration should have the lowest variance across experiments and time bins while maintaining one of the highest median test BSR. By this criteria, we see pre-computed, directed edges with PageRank centrality is the “best” CPE configuration.

### Comparing pathway selection methodologies

We perform two pathway selection experiments using two data sets: 1) 4 studies (the training data for the 4 to 2 and the 4 to 3 experiments) and 2) 6 studies (the training data for the 6 to 1 experiments).

In these experiments, we compare the pathways that are selected by the pathway expression methods to the pathways selected by standard pathway ranking algorithms, ORA and CePA, as well as a list of influenza related pathways (labeled Flu) from Reactome. We find this list of influenza related pathways by simply searching for influenza on the Reactome website. We do not expect high overlap with these influenza pathways since they are likely only activated at later time points during infection. For methodological consistency in these Jaccard plots, we use the same edge and centrality methods for CePa and CPE, namely pre-computed, directed edges with PageRank centrality.

**Figure 2:**
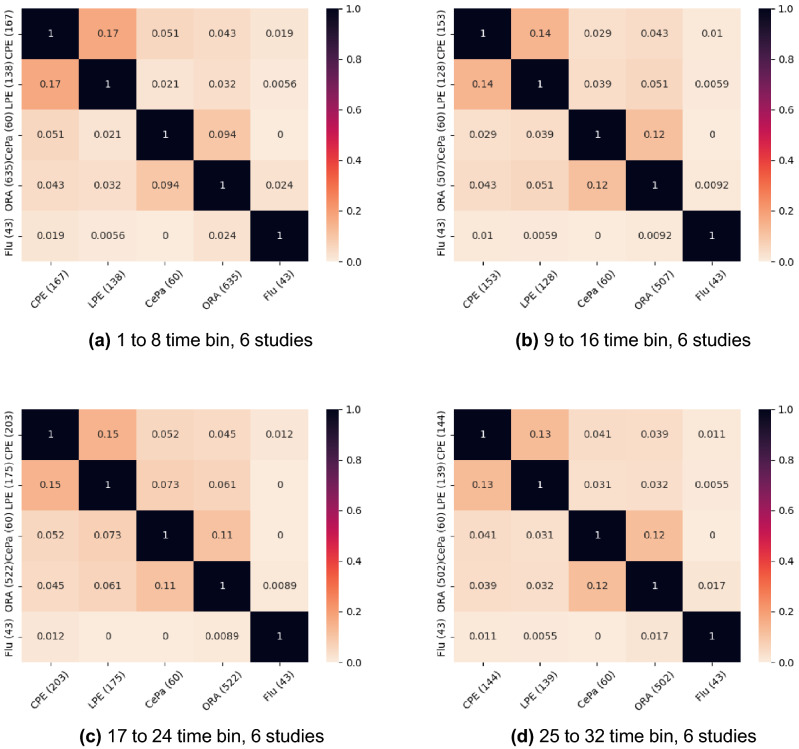
Jaccard overlap between the selected pathways for different methodologies. Pathways are selected using the 6 training studies. Each plot is for a different train/test experiment with LIMMA using subject identifier. The CPE configuration is pre-computed, directed edges with PageRank centrality.

For comparison, we use the Jaccard/Tanimoto similarity coefficient as a measure of overlap between these two sets of pathways. These comparisons for the 6 study features are shown in Fig. 2. The Jaccard similarity plot for the features from the 4 training study experiments are in Supplementary Information.

Over all methods and experiments, we notice LPE and CPE have a high Jaccard similarity, and CePa and ORA have a relatively high Jaccard similarity. CePa and ORA both have a small Jaccard similarity with LPE and CPE. We generally see low overlap between all methods and the influenza pathways. Generally, ORA is the method with the highest overlap with the influenza pathways since it detects at least twice as many pathways as each of the other pathway selection methods. In fact, CPE has absolutely no overlap with influenza pathways for the 4 study experiments. In contrast, at the 6 study experiments, we see that CPE has a higher overlap with the flu pathways than all other methods at the 9 to 16 and 17 to 24 time bins even though it detects less than half as many pathways as ORA. LPE has the highest overlap with the flu pathways at the 1 to 8 hour time bin for the 4 study experiment even though there are only 60 LPE pathways in contrast to the 254 ORA pathways.

Next, we investigate the robustness of these selected pathways across different studies in Table 2. To do this, we look at the Jaccard overlap between the pathways from 4 training studies and those from 6 training studies for each method and time bin. We find that pathways found by ORA are the most robust to this change in training data. Generally, the overlap between the pathway expression pathways is half of that from ORA. The CePA overlap is far smaller than the other methods and generally 10 times smaller than the overlap from ORA.

**Table 2:** Jaccard overlap between the selected pathways across the 4 study and the 6 study pathways LIMMA was used to normalize the data for subject identifier.

Method	Time bin	Jaccard overlap	4 studies	6 studies
CPE	1–8	0.0877	81	167
LPE	1–8	0.0588	60	138
CEPA	1–8	0.0169	60	60
ORA	1–8	0.2095	254	635
CPE	9–16	0.1014	75	153
LPE	9–16	0.0885	81	128
CEPA	9–16	0.0169	60	60
ORA	9–16	0.2288	227	507
CPE	17–24	0.1408	113	203
LPE	17–24	0.1186	89	175
CEPA	17–24	0.0435	60	60
ORA	17–24	0.3574	363	552
CPE	25–32	0.0846	74	144
LPE	25–32	0.1196	67	139
CEPA	25–32	0.0256	60	60
ORA	25–32	0.2222	235	502

The final two columns are the number of pathways selected by the method using the stated training dataset (4 or 6 studies). The CPE configuration is pre-computed, directed edges with PageRank centrality.

### Top CPE pathways

**Figure 3:**
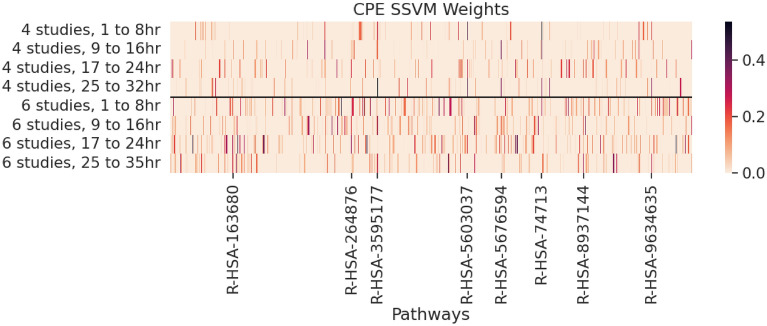
SSVM weights for the 4 and 6 study training datasets by pathway. The CPE configuration with pre-computed, directed edges/ PageRank centrality.

So far, we have seen that CPE generally produces the highest classification rates in our experiments over all methods examined in Table 1. For this entire section we will be using the CPE configuration with pre-computed, directed edges/ PageRank centrality. We use the SSVM weights on the training data to determine the top pathways for each experiment. Figure 3 is a heatmap of these SSVM weights for the union of the selected pathways from each experiment and time bin. The most discriminatory pathways on the training data are the pathways with the highest SSVM weights. We notice that some pathways remain activated across all times and studies. This is indicated by a dark streak in one column which persists across all rows.

The top pathways from CPE across all experiments are found by adding the SSVM weights across all experiments and time bins are in Table 3. This amounts to adding the values of each column in Fig. 3 to compute a pathway score for each selected pathway. A plot of these sorted scores is in the Top CPE Pathways subsection of the “Results” section in Supplementary Information and is used to determine a total SSVM weight threshold of 0.7 to identify the pathways listed in Table 3. A brief discussion of the relationships between the pathways in Table 3 and influenza appears in the “Discussion” section.

**Table 3:** The pathways with the highest magnitude SVM weights summed over all experiments and times.

Reactome ID	Pathway name
R-HSA-163680	AMPK inhibits chREBP transcriptional activation activity
R-HSA-264876	Insulin processing
R-HSA-8937144	Aryl hydrocarbon receptor signalling
R-HSA-5676594	TNF receptor superfamily (TNFSF) members mediating non-canonical NF-kB pathway
R-HSA-9634635	Estrogen-stimulated signaling through PRKCZ
R-HSA-5603037	IRAK4 deficiency (TLR5)
R-HSA-74713	IRS activation
R-HSA-3595177	Defective CHSY1 causes TPBS

CPE configurations are pre-computed, directed edges with PageRank centrality. All experiments apply LIMMA using subject identifier to the data.

Now we present the top pathways from CPE for each experiment in Table 4. We define these top pathways as those pathways with the highest SSVM weight in the feature selection process for their experiment and time bin. We notice that R-HSA-74713 appears in two time bins for the 4 study experiment. From our searches on Reactome, none of these pathways are directly labeled as influenza pathways. However, the Jaccard overlap heatmap in Fig. 2 suggests that the pathways selected using this CPE configuration contains some of the influenza virus signal because they have the highest overlap with the influenza pathways out of all pathway selection techniques at the 9 to 16 and 17 to 24 hour time bins. Therefore, we suggest investigation into R-HSA-8939242 and R-HSA-9694631 pathways and their relationship to respiratory viruses. Additionally, R-HSA-3595177 and R-HSA-74713 appear in the both Tables 3 and 4 and therefore we suggest further research into the connection between the R-HSA-3595177 and R-HSA-74713 pathways and respiratory viruses.

**Table 4:** The pathways with the highest magnitude SVM weights from CPE for each experiment and time.

Experiment	Time (h)	Reactome ID	Pathway name	Genes	Probes
4 studies	1-8	R-HSA-74713	IRS activation	5	10
4 studies	9-16	R-HSA-74713	IRS activation	5	10
4 studies	17-24	R-HSA-2179392	EGFR Transactivation by Gastrin	9	22
4 studies	25-32	R-HSA-3595177	Defective CHSY1 causes TPBS	8	21
6 studies	1-8	R-HSA-2485179	Activation of the phototransduction cascade	11	20
6 studies	9-16	R-HSA-8939242	RUNX1 regulates transcription of genes involved in differentiation of keratinocytes	8	25
6 studies	17-24	R-HSA-9694631	Maturation of nucleoprotein	16	27
6 studies	25-32	R-HSA-5218921	VEGFR2 mediated cell proliferation	21	53

CPE configurations are pre-computed, directed edges with PageRank centrality. All experiments apply LIMMA using subject identifier to the data.

## Discussion

In the results we provided a comparison between pathway expression and gene expression methods with the same linear feature selection and classification methodology on train/test partitions of the GSE73072 data set. Both methods selected features (pathways or genes) using influenza training data then use these selected features in a LOSO (Leave One Subject Out) cross validation experiment with an SVM classifier on testing data. In these experiments, we found that pathway expression (CPE and LPE) methods generally produce higher test BSRs than gene expression methods. Specifically, we found that pathway expression produced higher test BSR than gene expression on 10 out of 12 classification experiments.

We used the distributions of test BSRs across experiments and time bins to conclude that pre-computed, directed edges with PageRank centrality is the “best” centrality configuration. We found that CPE with pre-computed edges and PageRank centrality produced the highest test BSR out of all CPE configurations for most experiments and time bins. Consequently, we reported the pre-computed, directed edges with PageRank centrality CPE configuration in all our classification rates and pathway ranking comparisons.

In addition to the feature selection and classification experiments, we compared these selected pathways from pathway expression methods to two standard gene expression pathway analysis methods: CePa and ORA. We found that the pathways selected from pathway expression methods generally have little similarity to pathways from these gene expression methods. This suggests that the pathway expression methods along with SSVM feature selection provides a unique pipeline that selects discriminatory pathways which are not detected by standard pathway analyses on gene expression data.

Pathway expression methods are also pulling out some respiratory virus signal since LPE produced a non-zero overlap with influenza pathways on 6 out of 8 experiments and time bins and CPE (with pre-computed, directed edges and PageRank centrality) had a non-zero overlap with the influenza pathways for each time bin in the 6 study experiment while sporting the highest overlap in 2 out of these 4 time bins. Since CPE has a non-zero overlap with the influenza pathways for the each of the time bins in the 6 study experiments, we suggest investigation into the links between respiratory viruses and the top pathways from this method, R-HSA-2485179, R-HSA-8939242, R-HSA-9694631 and R-HSA-5218921. The pathways R-HSA-8939242 and R-HSA-9694631 have the most promise to be interesting since they were the top pathways at the time bins where CPE had the highest overlap with the influenza pathways out each one of the tested methods.

When we look at both the highest magnitude SVM weight pathways from CPE by experiment and overall, we see that R-HSA-74713 and R-HSA-3595177 appear in both lists. Therefore these are the most discriminatory of the pathways listed in the Results section. In fact, the R-HSA-74713 pathway appeared in more than one experiment and time bin. Our methods detected two insulin-related pathways: R-HSA-74713 and R-HSA-264876. R-HSA-74713 is a mediator of insulin signaling events and R-HSA-264876 is an insulin processing pathway. Insulin signaling is related to the influenza virus because the influenza virus impairs insulin signaling and down-regulates the expression of genes in the insulin pathway which supports our detection of the^28,29^. Additionally, R-HSA-3595177 is involved in the synthesis of chondroitin sulfate which has been shown to be involved with pulmonary immune response to influenza infection by Brune et al.^30^.

Many of the other pathways found to have the highest total SSVM weights across all experiments and time bins have links to the influenza virus. The R-HSA-163680 pathway involves AMPK signaling which is known to be significant in the modulation of viral infections^31^. Aryl hydrocarbon receptor (ARH) signaling (the R-HSA-8937144 pathway) is important part of the immune system, and ARH specifically regulates the immune response which is directly related to infection^32,33^. We find that the R-HSA-5676594 pathway is related to NF-$\kappa$B which is known to activate during RSV infection, especially early during the infection^34,35^. The R-HSA-9634635 pathway involves signaling through PRKCZ and it was found in one study that suppressing the expression of PRKCZ reduces RSV infection which suggests that the two are linked to some extent^36^. The R-HSA-5603037 pathway is involved with IRAK4 deficiency. Kim et al. found that IRAK4 kinase activity is suggested be involved with TLR-dependent immune responses and influenza virus is dependent on IRAK4 kinase activity^37^.

Not only does our implementation of pathway expression produce interesting pathways, it also is robust to perturbation in theory and in practice. Linear pathway expression for a given pathway, is robust to perturbation of the gene expression levels in a pathway by mean 0 noise because it is a mean of gene expression levels in a pathway. Centrality pathway expression is only approximately robust to such an addition of noise since it is a weighted mean of gene expression levels. In our experiments we found pathway expression methods proved to be more robust to subject batch effect than gene expression methods. Linear pathway expression was the pathway expression method that was the least affected by subject batch affect. The plot to support this claim is in the Results section in the Supplementary Information.

We also examine the robustness of pathway expression with regards to the pathways selected using SSVM feature selection on pathway expression data. We do this by comparing these selected pathways to those selected using gene expression features along with standard pathway ranking methods. Specifically, we look at the overlap between the pathways found with 4 studies and those found with 6 studies. We find that the pathways selected by ORA have the highest overlap, the pathway expression method have the second highest and CePa has the lowest overlap. It is expected that this overlap is somewhat proportional to the number of pathways that were selected by each method since we are sampling from a background set of pathways. In an extreme example, if the each of the two pathway sets have more than half of the total pathways, then they must overlap. We see this proportionality of the number of pathways selected and the overlap for each of the methods. Additionally, the viruses in the 4 study training dataset are H3N2 and H1N1), and the viruses in the 6 training dataset are H3N2, H1N1, RSV and HRV. We note that the relatively high overlap in the pathways detected by ORA indicates that this method pulls out the general respiratory virus signal but does not detect the differences between the different viruses in the training datasets. On the other hand, the low overlap for CePa suggests that this method detects the difference between the datasets but does not capture the overall respiratory virus signal. Pathway expression methods take the middle ground since their overlap is between the standard pathway analysis methods (ORA and CePa). That is to say, the pathways found using pathway expression encapsulate the difference between the viruses in the training datasets while still maintaining the overall respiratory virus signal.

Returning to analysing our methodologies, CPE and LPE are simple linear models for translating gene expression data to pathway expression data which result in improved BSR of our tested machine learning models. The linear nature of our implementation of pathway expression, especially LPE, makes it possibly one of the simplest methods available for transforming vectors from the gene space to the pathway space. A different non-linear pathway expression formulation may improve upon the results with pathway expression presented in this paper. One non-linear modification of LPE has been done by using the absolute values of the entries in the gene expression matrix instead. This modification collapses the gene expression data into the positive hyper-octant and results in a loss of information. However, there are many possible variants of non-linear pathway expression that require further investigation.

The pathway expression methods in this paper are meant to re-inspire a wide variety of investigations into the pathway expression pipeline including batch correction, pathway expression generation and downstream pathway expression analyses. Investigation into the robustness of pathway expression methods to batch effects could be done by running experiments with corrections for study or strain effects. In this paper, we applied batch normalization to gene expression vectors before calculating pathway expression. Another interesting experiment in future work could be to apply batch normalization to the pathway expression vectors themselves. Within the framework of CPE, different methods for network generation, and centrality measures still need to be tested. LPE and CPE are arguably the simplest pathway expression methods since they are effectively an averaging of gene expression levels. From a bird’s eye view, pathway expression methods are any method to transform a gene expression matrix to a pathway expression matrix. Therefore any type of pathway transition matrix, as well as a non-linear transform, can be applied to gene expression data to produce pathway expression data. This leaves the door wide open to the use of other central prototypes for generating pathway expression matrices. Pathway expression data can be seen as just a pre-processing step. Hence, any machine learning algorithm that has been used on gene expression data can be used on pathway expression data to select pathways, classify pathways, cluster pathways, etc. Hence, the opportunity for novel work with pathway expression can include an investigation into pathway expression pre-processing, batch correction techniques, methods for pathway expression generation, and downstream pathway expression analyses using statistics, machine learning algorithms and more. The bottom line is, creativity and/or applying sound biological principles while designing a pathway expression workflow will be key in selecting meaningful pathways and perhaps improve classification rates.

Our 4 to 2, 4 to 3 and 6 to 1 classification experiments in the results are designed to mimic the experiments that were done by Aminian et al.^3^. For a direct comparison of best CPE results between this paper to the gene expression results using SVM from Aminian et al. see Table 5. Our results with LPE, CPE *and GE* generally produce lower SVM BSRs than those from the same experiments in Aminian et al. This is not surprising since the feature selection technique in this paper is far more simple and less robust than what was done by Aminian et al. However, at the 25 to 32 hour time bin, the pathway expression methods produce higher BSR than the classification results in Aminian et al. by 1 to 4 percent in BSR in two out of three experiments. The higher classification BSRs at the latest time bin are consistent with the concept that genes generally don’t work in concert in pathways during the early hours after infection. A PCA of all the probes related to genes in immune response pathway, $\alpha /\beta$ Interferon, by Aminian et al. highlights the inactivity of the pathway during early hours of infection and activation of the pathway during later hours after infection. Perhaps by running experiments at later time bins and/or using the same feature selection technique that was used by Aminian et al. we’d see that these novel pathway expression methods produce even higher BSRs. Additionally, any biologically informed modification of the pathway expression pipeline presented in this paper could increase test classification rates while detecting even more biologically informative pathways. We choose to include this table and it’s analysis for completeness and to inspire future work on improving pathway expression transformations and optimization of downstream ML feature selection and classification architectures with CPE and LPE.

**Table 5:** Classifications balanced success rates of SVM in a LOSO experiment on test data across different experiments within 32 hours after infection.

Time bin	Paper	4–2	4–3	6–1
1–8	This paper	80.04	73.45	$\mathbf {89.44}$
1–8	Aminian et al.	$\mathbf {84.74}$	$\mathbf {82.06}$	87.97
9–16	This paper	82.13	80.84	89.44
9–16	Aminian et al.	$\mathbf {93.21}$	$\mathbf {90.37}$	$\mathbf {100.00}$
17–24	This paper	77.07	77.27	80.35
17–24	Aminian et al.	$\mathbf {81.58}$	$\mathbf {78.82}$	$\mathbf {86.36}$
25–32	This paper	$\mathbf {92.49}$	82.83	$\mathbf {90.91}$
25–32	Aminian et al.	88.21	$\mathbf {85.46}$	89.44

The highest values for each experiment and time bin are in bold.

All experiments in this table use LIMMA normalization on subject identifier. The majority of the best results from this paper are using CPE.

Overall, the pathway expression analysis framework developed in this work provides a concise approach for characterizing the biological processes associated with the host response to infection. Although we produce lower classification rates than those in Aminian et al, in a head to head comparison with gene expression, we see that CPE and LPE improve classification rates. Moreover, we envision that pathway selection using this approach may provide additional insights into biological mechanisms associated with the host response to infection. The pathways detected using CPE are connected to the immune response and to some specific respiratory viruses. Our preliminary experiments addressing time-evolving human clinical data provide some insight into the pathway activity for humans infected with respiratory viruses. Finally, we offer a python package for computing CPE, LPE and CePa https://github.com/nmank/PathwayAnalysis.

## Methods

In this section we detail the models, metrics, experiments, and data sets used in this paper. We run two mirrored classification and pathway selection pipelines on pathway expression data and gene expression data.

Our pipeline used in the pathway expression (CPE and LPE) classification experiments is the following:

1. Batch normalize via LIMMA each train and test partition separately by subject ID.
2. Select pathways, viewed as features, in pathway expression data using the training data (LPE-SSVM or CPE-SSVM).
3. Run a LOSO classification experiment using SVM on test pathway expression data restricted to the selected pathways (generated by LPE-SSVM or CPE-SSVM) and record mean test statistics (eg. BSR).

For pathway selection using pathway expression we simply use the pathways selected by SSVM on the training data and rank these selected pathways by their SSVM weights.

The pipeline for the GE classification experiments is:

1. Batch normalize via LIMMA each train and test partition separately by subject ID.
2. Select genes, viewed as features, using the training data using SSVM.
3. Run a LOSO classification experiment using SVM on test gene expression data restricted to the selected genes and record the mean test statistics (eg. BSR).

For pathway selection using gene expression data we use the genes selected by SSVM on the training data as input ORA or CePa pathway selection. ORA is implemented using the *p*-value from the analysis.identifiers function reactome2py, (https://github.com/reactome/reactome2py). CePa is implemented in the GLPE.simple_transform function in the PathwayAnalysis package (https://github.com/nmank/PathwayAnalysis). Details on the ORA and CePa methodologies are provided in Pathway Ranking Using Gene Feature Sets in the Methods section of the Supplementary Information.

### Data set (GSE73072)

In this paper we perform experiments on the GSE73072 data set^2^ from the NCBI Gene Expression Omnibus (GEO). This data set is a microarray gene expression data set for human subjects challenged with the influenza virus. These data were collected from 7 studies by Duke, UVA and hVIVO and was funded by the Defense Advanced Research Projects Agency (DARPA). The entire data set consists of 22277 probe identifiers and 148 human subjects infected with four different types of respiratory viruses: HRV, RSV, H1N1 and H3N2. The data samples are collected at irregular time intervals from 38 hours before infection to 680 hours after infection. The data can be found here https://www.ncbi.nlm.nih.gov/geo/query/acc.cgi?acc=GSE73072.

We run two binary classification experiments with GSE73072 data set that are copies of experiments by Aminian et al.^3^. In these experiments we classify between control subjects and shedding subjects. Our control group consists of all subjects between, and including, 38 to 0 hours before infection. We define (early) shedders as pre-symptomatic subjects within 32 hours after infection who will eventually be characterized as shedders over the course of the immune response. We break our data sets down further into 4 sets of shedders from evenly spaced time bins within 32 hours after infection. Our train/test splits are described in Supplementary Information using the format: study identifier (virus).

For data pre-processing, we perform two steps which follow the experimental design of Aminian et al. so that we can make a faithful comparison of classification rates^3^. First, we normalize the entire data set using the robust multi–array average (RMA)^38^ method. We then correct for subject differences by applying normalization across subject identifier using the LIMMA package^39^ to each train/test partition separately.

We use the Reactome Pathway Database^9^ to determine pathway membership and networks. We use the R package graphite^40^ to generate pathway networks with edges. The graphite package, using the information from Reactome, gives protein-based edges which are then translated using Entrez gene identifiers. We also directly use the Reactome database information, available on the Reactome website, to ensure genes are in appropriate pathways and edges are within Reactome pathways only (any edges outside of a pathway are removed before analysis).

The GSE73072 data set is a microarray data set with features given as microarray probe identifiers (probe IDs). We choose to do all our analyses on the probe IDs rather than determine a mapping to Entrez identifiers (Entrez IDs) in order to retain as much feature information as possible. However, this means that the pathway membership and network edge information needs to be converted to probe IDs. We use the following mapping from Entrez ID pathway networks to probe ID pathway networks. Although this method results in a loss of some information, *we retain all the probe IDs that correspond to Entrez IDs from in affymetrix platform file*. The edges between probe IDs derived from this method are used for the edges and edge weights for the pre-computed directed and undirected edges for CPE and CePa workflows.

1. For every probe ID in affymetrix platform file, map to the first Entrez ID it its associated list of Entrez IDs (in the event of multiple maps). Since some probe IDs are not mapped to Entrez IDs via the platform file, we loose $37.1\%$ of the probe IDs in the original data set.
2. Use the pathway network information to draw an Entrez ID network
3. Map all the Entrez ID nodes from the pathway networks to probe IDs using this mapping. *Note: this means some nodes are mapped to multiple probe IDs.* Assign edges in the pathway networks with probe ID nodes according to the edges between Entrez IDs. Multiple probe IDs that map to the same Entrez ID will have no edges between them. Probe IDs with no Entrez ID to map to are dropped. Entrez IDs with no probe IDs mapping to them are dropped.

**Figure 4:**
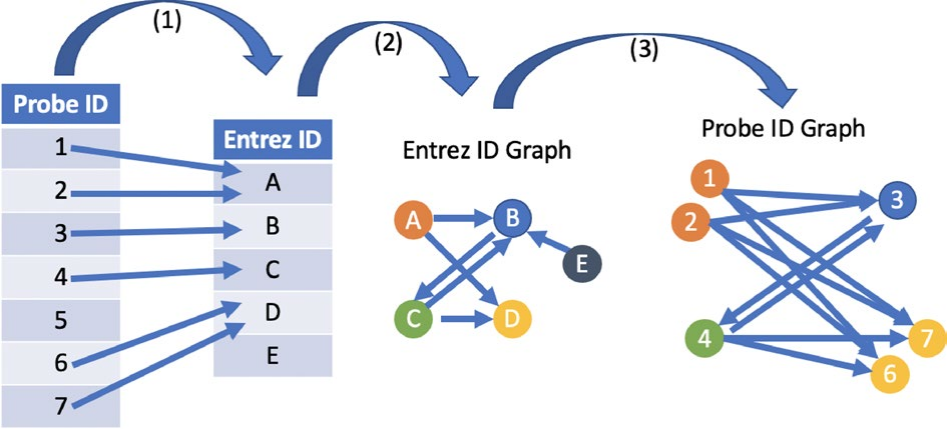
How we generate Probe ID pathway networks using the platform file and Entrez IDs pathway networks.

For a visual of this mapping see Fig. 4.

### Pathway expression (LPE and CPE)

Pathway expression is a method to represent biological data as a pathway expression matrix with pathway expression levels as features. In this section we develop the mathematical underpinnings of the pathway expression methods used in this paper.

Let $\textbf{X}\in \mathbb {R}^{p \times n}$ be a GE matrix with *n* gene expression levels for *p* subjects. In a gene expression matrix each gene is assigned a gene index and each subject is assigned a subject index. We can map $\textbf{X}$ to a “pathway expression matrix” $\textbf{Y}\in \mathbb {R}^{p \times m}$ with *m* pathway expression levels for *p* subjects. In a pathway expression matrix each pathway is assigned a pathway index and each subject is assigned a subject index. We define such a linear mapping in 1 using the pathway transition matrix $\textbf{P}\in \mathbb {R}^{n \times m}$.

1 $$\begin{aligned} \textbf{Y}= \textbf{X}\textbf{P}\end{aligned}$$

Let $P^{(i,j)}$ be the entry in the *i*th row and *j*th column of $\textbf{P}$. The most simple definition of $\textbf{P}$ is pathway membership in Eq. (2) and is used to compute a linear pathway expression (LPE) matrix.

2 $$\begin{aligned} P^{(i,j)} = {\left\{ \begin{array}{ll}1 &{} \text { if gene }i\text { is in pathway }j\\ 0 &{} \text { otherwise.}\\ \end{array}\right. } \end{aligned}$$

A slightly more involved definition of pathway expression is centrality pathway expression (CPE) where we weight gene expression levels by their centrality within a pathway network. This pathway transition matrix is denoted $\textbf{P}_c$ and called the pathway centrality transition matrix. We construct $\textbf{P}_c$ using the centrality of the gene *i* in the *j*th pathway network $c_{j}(i)$ in Eq. (3) where $\Vert \textbf{c}_{j} \Vert _1 = \sum _{i=1}^n |c_{j}(i)|$.

3 $$\begin{aligned} P_c^{(i,j)} = {\left\{ \begin{array}{ll}\frac{c_{j}(i)}{ \Vert \textbf{c}_{j}\Vert _1} &{} \text { if gene }i\text { is in pathway }j\\ 0 &{} \text { otherwise.}\\ \end{array}\right. } \end{aligned}$$

**Figure 5:**
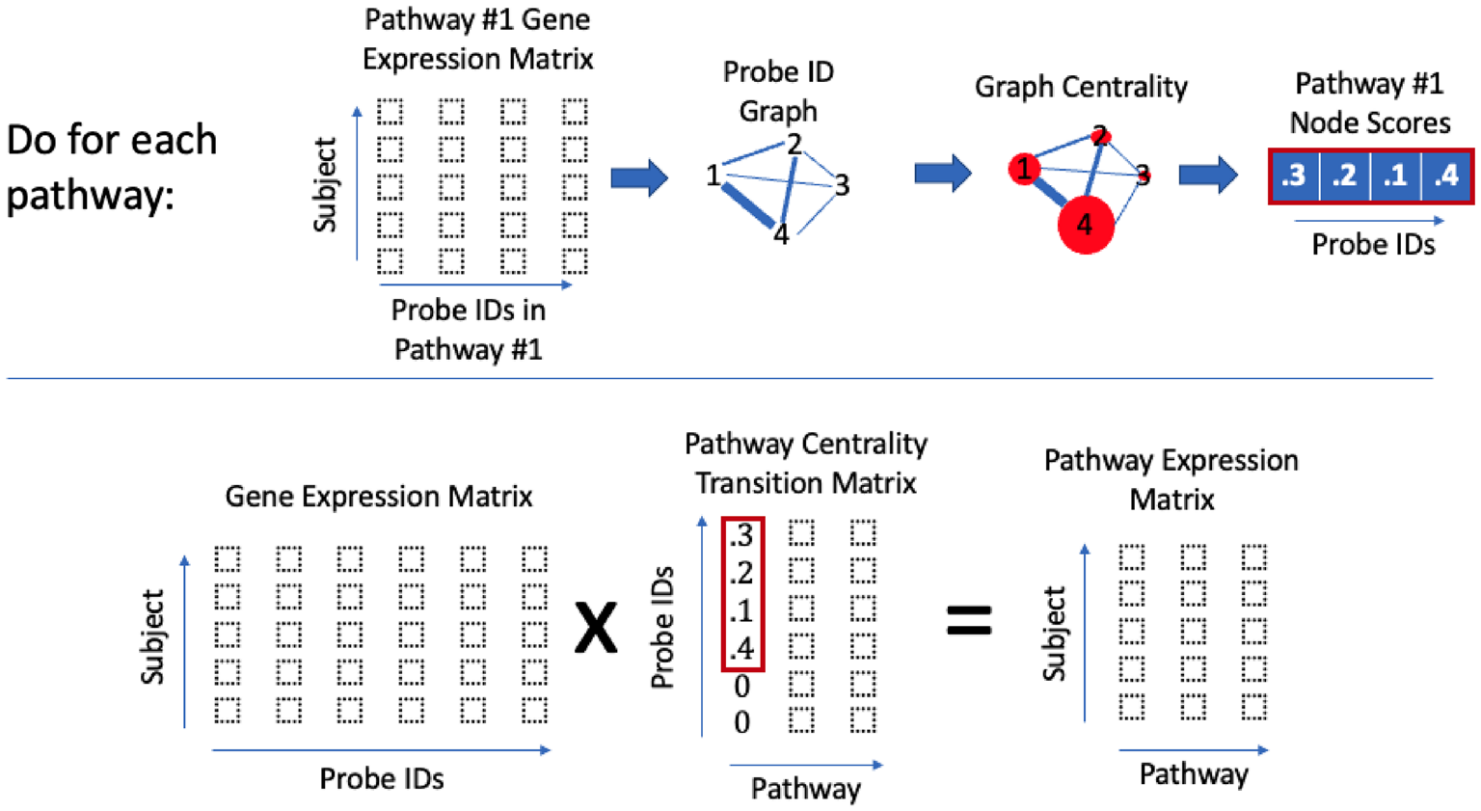
The workflow for CPE.

The full CPE algorithm is in Fig. 5. For our implementations of CPE data sets, we use the same 6 combinations of pathway network edges and centrality measures that are used in CePa: pre-computed directed, pre-computed undirected or correlation edges with either out-degree (normalized by maximum out-degree) or PageRank centrality methods.

Now we will investigate CPE further by formulating the calculation of pathway expression as an optimization problem. Let $\textbf{Y}= [\textbf{y}_1, \textbf{y}_2, \dots , \textbf{y}_m]$ where $\textbf{y}_i \in \mathbb {R}^p$. We call $\textbf{y}_j$ the pathway expression vector for pathway *j*. Analogously, let $\textbf{X}= [\textbf{x}_1, \textbf{x}_2, \dots , \textbf{x}_n]$ where $\textbf{x}_i \in \mathbb {R}^p$ where we call $\textbf{x}_i$ the gene expression vector for gene *i*. We claim

4 $$\begin{aligned} \textbf{y}_j = \arg \min n \sum _{i = 1}^n P^{(i,j)}\Vert \textbf{z} - \textbf{x}_i \Vert _2^2. \end{aligned}$$

The obvious solution to this problem is the scaled average of the set $\{P^{(i,j)} \textbf{x}_i \}_{i =1}^n$. But notice that this is exactly

5 $$\begin{aligned} \textbf{y}_j = \sum _{i=1}^p P^{(i,j)} \textbf{x}_i. \end{aligned}$$

Doing this for every *j* we recover Eq. (1).

This outlines a methodology for translating a data set from the gene space to the pathway space using types of pathway expression. The LPE and CPE methods used in this paper can be found at on GitHub in the PathwayAnalysis repository https://github.com/nmank/PathwayAnalysis. The code in this repository can be used as an out of the box method for pathway expression analysis on other data sets. We can now leverage our data sets in the pathway expression matrix format to determine discriminatory pathways for a classification problem.

### SSVM feature selection

In this paper we use SSVM feature selection with gene and pathway expression matrices to find the best genes and pathways. This is done by running SSVM on the training data, then selecting features based on the SSVM weights. For simplicity, the SSVM feature selection in our experiments is limited to a rendition of the first iteration of iterated feature removal (IFR) from O’Hara et al.^6^. Throughout this section features can be genes or pathways depending on whether the experiment uses gene expression or pathway expression.

Our feature selection methodology starts with all the *m* features $P_1,P_2,\dots , P_m$ ordered with respect to their corresponding magnitude of their SSVM weights $w_1 \ge w_2 \ge \dots \ge w_m \ge 0$ calculated on whatever classification experiment we are analyzing. Our goal is to take only the features that have significant weights from the SSVM model. To determine which weights are significant, we calculate the weight ratios $r_i = w_{i-1}/w_{i}$ and look for a “jump”, that is, we find where the weights rapidly decrease for the first time over a certain threshold. This will be reflected in the ratios as a large “jump” in value. For our experiments we set our “jump” ratio at 5. After we have isolated the top features by weight, we then add in the features that are at least .9 correlated to these features using their training data (either GE, LPE or CPE depending on the experiment). The number of added correlated features changes depending on the initial feature set.

### Evaluation

For comparison between sets of pathways, we use the Jaccard/Tanimoto similarity coefficient. Given two sets of pathways, *P* and $P'$, the Jaccard/Tanimoto similarity coefficient between these two sets is defined in Eq. (6).

6 $$\begin{aligned} \frac{|P \cap P'|}{|P \cup P'|} \end{aligned}$$

We evaluate the novel linear pathway expression data by using SVM to classify between controls and shedders in a LOSO experiment on the test data set using the only the pathways that were selected on the training data. We use the mean test BSR, precision, recall and accuracy of these SVMs to determine the “best” method.

We compute mean test BSR for these LOSO experiments on test data in three steps. (1) Compute a confusion matrix for each subject’s SVM experiment on the test data. (2) Sum all the subject confusion matrices. (3) Compute the average of the true positive rate and the true negative rate. This metric provides a better model assessment than accuracy on data sets with imbalanced class sizes. Precision, recall and accuracy are computed in a similar manner leveraging the sum of all the confusion matrices across experiments. *Note: that standard deviation for these statistics is not available since we are computing them from a sum of the confusion matrices across the LOSO experiments.*

These pathway expression results are compared to results using the same workflow on gene expression data. We run an SSVM feature selection on the training gene expression matrices to select discriminatory genes. Then we restrict the gene expression matrices of the test data to the discriminatory genes and run a LOSO SVM experiment on the test data set and use the BSR to compare against the BSRs from the LPE methods.

## Supplementary Information

Supplementary Information.

## Data Availability

The data sets analysed during the current study are available on NCBI, https://www.ncbi.nlm.nih.gov/geo/query/acc.cgi?acc=GSE73072. The code for the experiments in this paper is available in GitHub in the PathwayAnalysisPaper repository https://github.com/nmank/PathwayAnalysisPaper.
