# Supplementary material for: Pathway expression analysis
Source: Sci Rep. 2022 Dec 17;12:21839. doi: 10.1038/s41598-022-26381-x (PMC9759056; doi:10.1038/s41598-022-26381-x)
Supplement: Supplementary file 1 — Supplementary Information. [file 41598_2022_26381_MOESM1_ESM.pdf]

Supplementary Information for Pathway Expression Analysis

Nathan Mankovich[1,*], Eric Kehoe[1,o], Amy Peterson[1,x], and Michael Kirby[1,+]

[1]Colorado State University, Mathematics, Fort Collins, 80523, USA
[*]nathan.mankovich@gmail.com
[o]ekehoe@colostate.edu
[x]apete41lsu@gmail.com
[+]michael.kirby@colostate.edu

ABSTRACT

This is the Supplementary Information for the Pathway Expression Analysis paper.

Results

This is the supplementary material for the Results section.

Visualization

For our PCA (Principle Component Analysis) visualization, we use gene expression data from 9 to 16 hours after infection that have been batch corrected for subject identifier using LIMMA. We select features using 4 studies containing H1N1 and H3N2 strains of influenza. Then we do two PCA plots of the first two principle components of data from 2 HRV test studies. One plot uses all the features for the PCA. The second plot uses only the features found using the 4 H1N1 and H3N2 studies. The feature selection is done with gene expression data and with pathway expression data using the same SSVM feature selector methodology. The object of this experiment is a head to head comparison between gene expression to pathway expression using the linear separation between between the controls (subjects before infection) and shedders (subjects from 9 to 16 hours after infection). These PCA plots are in Figure S1. For this experiment and time bin, we notice better linear separation between the pathway expression data than the gene expression data.

When we produce the same types of PCA visualizations using each train/test split, along with each of the different time bins and pathway expression types, we notice that the gene expression data appears to linearly separate better than pathway expression data. But, upon further investigation, we find that the explained variance ratio of the first two principle components for pathway expression data in these plots are generally less than 0.5. So most of the variance of pathway expression data is captured in the 3rd to n^{th} principle components. This provides some explanation for the poor linear separation of pathway expression data using the first two principle components of PCA.

0.1 Classification Results

CPE requires two parameters to be set: a pathway gene network edge type and a centrality measure. We test CPE by using either correlation edges or pre-computed (from Reactome) edges, which may be directed or undirected. For centrality measures, we use either PageRank or out-degree centrality. Table S1 is a table of the CPE configurations which produce the highest test BSR for each experiment. Using this table, we see there is no clear winning parameter combination for maximizing classification rates across all data partitions. However, we do observe that pre-computed edges with PageRank centrality is the most common method across all experiments and time bins to produce the highest BSR. In fact, at the 25 to 32 hour time bin the CPE parameters which produced the highest BSR are all undirected networks with pre-computed edges with PageRank centrality.

We now compare the effects of normalization via LIMMA on subject identifier on the classification results in Figure S2. The y-axis, Difference, is the difference in BSRs on the test studies with LIMMA normalized data for subject identifier and un-normalized data for subject identifier. LIMMA normalization using subject identifier increases the classification BSR for each method. When comparing LPE and CPE to GE, we notice that the inter-quartile range and median difference for pathway expression methods is smaller than those from the gene expression methods. This implies that the BSRs for gene expression methods change less uniformly and more on average from LIMMA batch correction on subject identifier than pathway expression methods. Consequently, we claim that pathway expression methods are more robust to subject differences

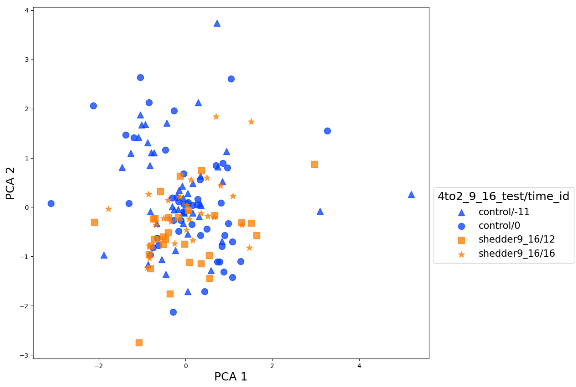

(a) GE test data with all the features.

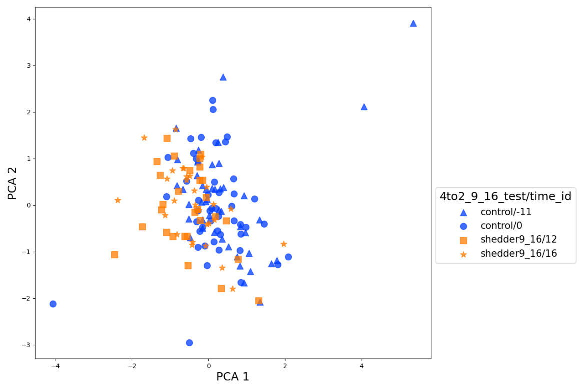

(b) GE test data with the selected features.

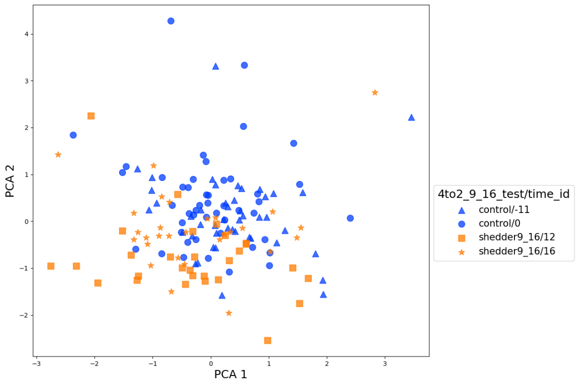

(c) CPE test data with all the features.

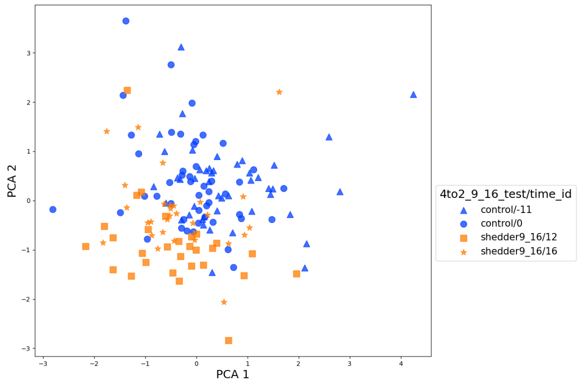

(d) CPE test data with the selected features.

Figure S1. A PCA embedding of the 2 HRV test studies using GE and CPE in the time bin 9 to 16 hours after infection. The first column is an embedding with all the features and the second is with the selected features. These CPE data are generated using pre-computed, undirected edges with out-degree centrality. The data have been batch corrected for subject identifier using LIMMA.

Experiment	Time Bin	Centrality	Similarity	Directed
4 to 2	1 to 8	PageRank	pre-computed	True
4 to 2	9 to 16	out-degree	pre-computed	False
4 to 2	17 to 24	out-degree	correlation	False
4 to 2	25 to 32	PageRank	pre-computed	False
4 to 3	1 to 8	out-degree	correlation	False
4 to 3	9 to 16	PageRank	pre-computed	False
4 to 3	17 to 24	out-degree	pre-computed	True
4 to 3	25 to 32	PageRank	pre-computed	False
6 to 1	1 to 8	PageRank	pre-computed	True
6 to 1	9 to 16	PageRank	correlation	False
6 to 1	17 to 24	PageRank	pre-computed	True
6 to 1	25 to 32	PageRank	pre-computed	False

Table S1. The CPE centrality and similarity configurations which resulted in the highest test BSR for CPE given each data partition and time bin. The data have been batch corrected for subject identifier using LIMMA.

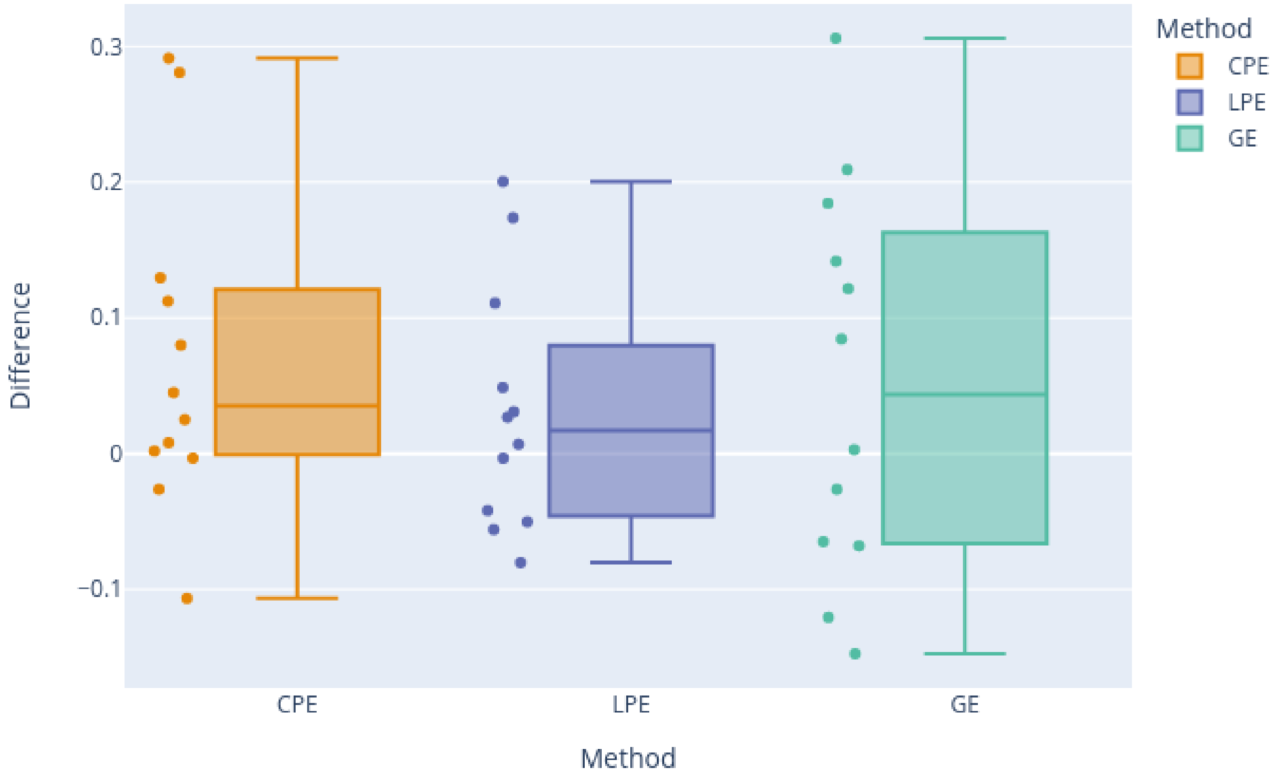

Figure S2. The difference between test BSR with LIMMA normalization using subject identifier and test BSR without LIMMA. Each box represents the distribution of these differences across experiment, time bin, and data partition for each method. A positive difference in BSR indicates that LIMMA normalization using subject identifier increased the classification accuracy. For CPE, we use pre-computed, directed edges with PageRank centrality.

within a class. In addition, linear pathway expression methods appear to be the most robust to subject differences within a class because the median difference for LPE in Figure S2 is nearer to 0 than the median difference for CPE and GE.

Comparing Pathway Selection Methodologies

We preform two pathway selection experiments using two data sets: 1)4 studies (the training data for the 4 to 2 and the 4 to 3 experiments) and 2) 6 studies (the training data for the 6 to 1 experiments).

In these experiments, we compare the pathways that are selected by the pathway expression methods to the pathways selected by standard pathway ranking algorithms, ORA and CePA, as well as a list of influenza related pathways (labeled Flu) from Reactome. We find this list of influenza related pathways by simply searching for influenza on the Reactome website. For methodological consistency in these Jaccard plots, we use the same edge and centrality methods for CePa and CPE, namely pre-computed, directed edges with PageRank centrality.

For comparison, we use the Jaccard/Tanimoto similarity coefficient as a measure of overlap between these two sets of pathways. The Jaccard similarity plot for the features from the 4 training study experiments are in Figure S3.

Top CPE Pathways

We use the sum of the SSVM weights of the selected pathways across all experiments to determine the top pathway. Figure S4 displays these sorted total SSVM weights by pathway. We notice that the weights increase exponentially and choose to take the 'best' pathways as those which are in steepest part of the curve. Therefore we take the top pathways across our experiments to have at least a total SSVM weight of more than .7. The list of these pathways appear in a table in the Top CPE Pathways

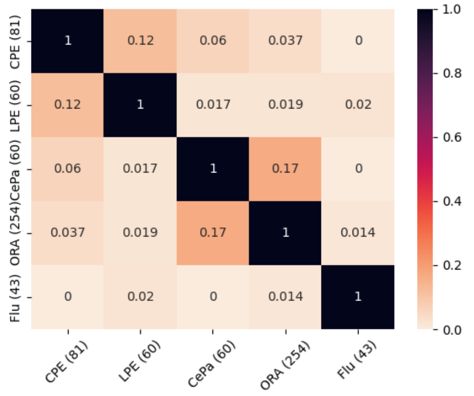

(a) 1 to 8 time bin, 4 studies

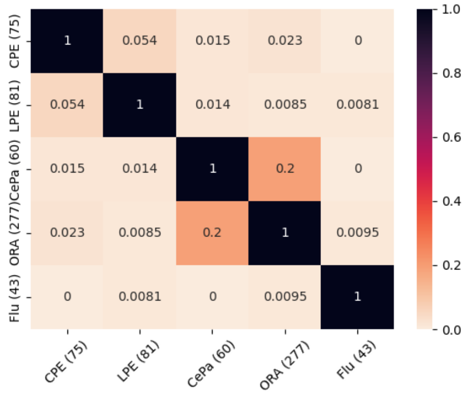

(b) 9 to 16 time bin, 4 studies

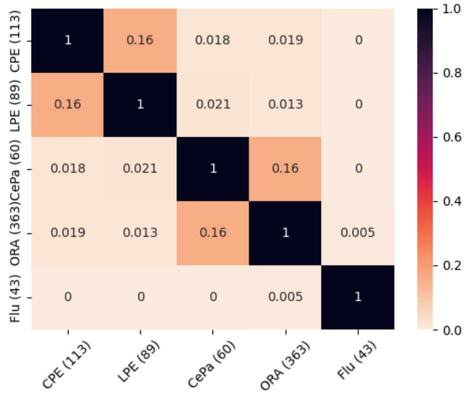

(c) 17 to 24 time bin, 4 studies

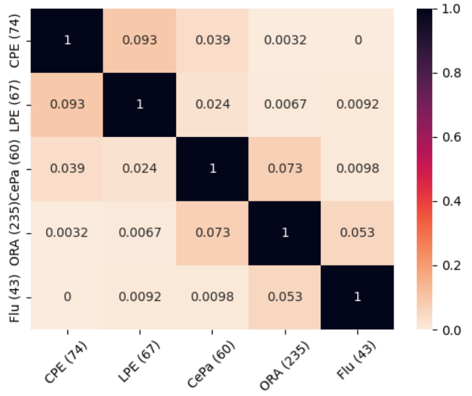

(d) 25 to 32 time bin, 4 studies

Figure S3. Jaccard overlap between the selected pathways for different methodologies. Pathways are selected using the 4 training studies. Each plot is for a different train/test experiment with LIMMA using subject identifier. The CPE configuration is pre-computed, directed edges with PageRank centrality.

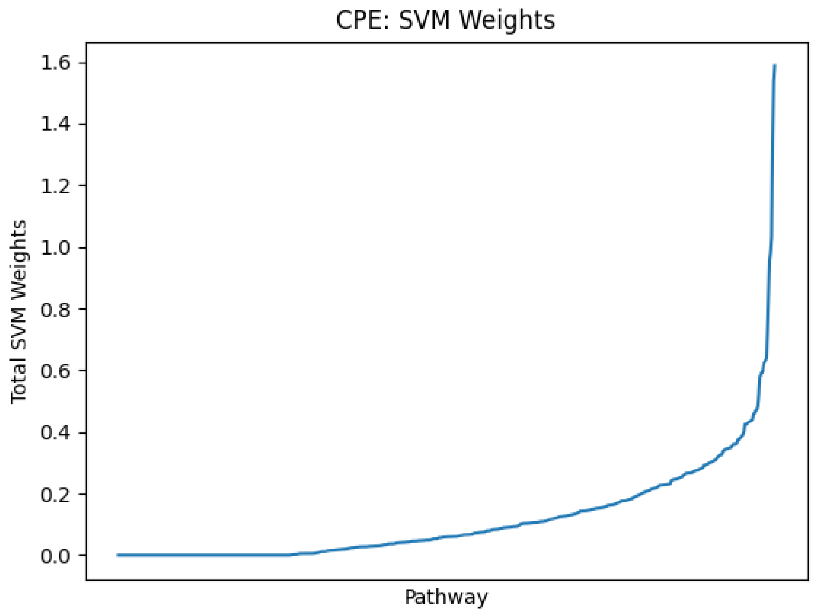

Figure S4. The sorted weights of the SSVM classifiers over all experiments and time bins for CPE. The CPE configurations in all experiments are pre-computed, directed edges with PageRank centrality.

Partition Name	Train	Test
4 to 2	DEE2 (H3N2), DEE3 (H1N1), DEE4 (H1N1), DEE5 (H3N2)	UVA (HRV), Duke (HRV)
4 to 3	DEE2 (H3N2), DEE3 (H1N1), DEE4 (H1N1), DEE5 (H3N2)	UVA (HRV), Duke (HRV), DEE1 (RSV)
6 to 1	UVA (HRV), Duke (HRV), DEE1 (RSV) DEE3 (H1N1), DEE4 (H1N1), DEE5 (H3N2)	DEE2 (H3N2)

Table S2. The train/test splits by study ID for the 4 to 2, 4 to 3 and 6 to 1 experiments. The parenthetical after each study ID is it's associated virus.

subsection of Methods in the manuscript.

Methods

This is the supplementary material for the Methods section.

Data Set (GSE73072)

We break our data sets down into 4 sets of shedders from evenly spaced time bins within 32 hours after infection. Our train/test splits are described in Table S2 using the format: study identifier (virus).

Pathway Ranking Using Gene Feature Sets

Let G be the set of all genes in the data set and $F \subseteq G$ be a feature set of genes. In this paper, the gene feature sets are calculated using SSVM feature selection (detailed in the SSVM Feature Selection section of the Methods section in the manuscript). In this subsection we will introduce two pathway ranking methods using a feature set of genes (or probe IDs): 1) ORA and 2) CePa. Let $P \subseteq G$ be the set of genes in a given pathway. These methods, ORA and CePa, assign a score to each pathway by leveraging the genes in F. The higher the score, the more important the pathway. In summary, each of these methods are a map $\phi : \mathcal{P} \to \mathbb{R}$ where $\mathcal{P}$ is the set of all pathways.

ORA is one of the most simple and widely used pathway scoring methods, so it is an ideal ground-truth pathway ranking method. Generally, ORA is a methodology for investigating the statistical significance of the overlap of genes in the feature set with known pathways. Using the hypergeometric distribution, ORA determines the p-value of the significance of the overlap, which is the score of the pathway, by

$$p = 1 - \sum_{k=0}^{f-1} \frac{\binom{\hat{F}}{k}\binom{N-\hat{F}}{n-k}}{\binom{N}{n}}.$$

In this formula, f is the number of genes in the overlap of the feature set and pathway, $\hat{F}$ is the number of genes in the feature set, N is the total number of genes possible, and n is the number of genes in the pathway. We implement this method using the Python package reactome2py to use the ORA analysis tools on the Reactome website. The GitHub page for reactome2py is https://github.com/reactome/reactome2py.

CePa is a network centrality informed method for pathway ranking on a given feature set, developed by Gu et al.[1]. This method combines the statistical notions that are used in ORA with biological pathway network information, specifically network centrality. For a given pathway P, we generate a pathway network where genes are the nodes. These edges are generated the same as those generated for CPE. Specifically, edges are either correlation between gene expression levels across subjects or pre-computed edges, specifically those edges generated from known biological connections using the graphite package[2].

Let $c_P : G \to \mathbb{R}$ be a pathway centrality map from the gene space to the real numbers. Given a gene $g \in G$, $c_P(g)$ is the centrality of g within it's pathway network. Then the rank for pathway P is just the sum of the centralities of the genes in both P and the feature set F as in Equation 1.

$$\text{CEPA}(P) = \sum_{n \in P \cap F} c_P(n) \tag{1}$$

We perform six CePa experiments where we use pre-computed directed, pre-computed un-directed or correlation edges with either out-degree (normalized by maximum out-degree) or PageRank centrality methods. To the best of our knowledge, this is the first time CePa has been used with PageRank centrality.

The final step in CePa is determining the pathway significance score relative to a collection of null feature sets. To do this, a large number of $m \in \mathbb{N}$ null trials are run with $|F|$ genes selected from a uniform distribution over all the genes. Then CePa is run for each of these null trials resulting in a set of null pathway rankings. Let the vector of the set of null pathway rankings for pathway P be denoted $\mathbf{n_P}$ and ordered by null feature set. Define the null value indicator map, denoted I, in Equation 2.

$$I(\mathbf{n_P}) = \begin{cases} 1 & \text{if } \mathbf{n_P} > \text{CEPA}(P) \\ 0 & \text{otherwise.} \end{cases} \tag{2}$$

We then use the indicator map to find the "null value" for pathway P as the mean of the entries in $I(\mathbf{n_P})$ in Equation 3.

$$\text{null value}(P) = \frac{\sum_{i=1}^{m} I(\mathbf{n_P})_i}{m} \tag{3}$$

Finally, the significant pathways from CePa are the pathways with the highest CePa scores and lowest null values. So given a pathway score threshold $\alpha \in \mathbb{R}$ and null value threshold $\varepsilon \in \mathbb{R}$, we say a pathway P is significant if $CEPA(P) > \alpha$ and null value$(P) < \varepsilon$. For this paper, we take the top 60 pathways with null value less than .05.

Overall, CePa consists of three steps. First determine pathway scores, then find null values and select pathways using pathway score and null value thresholds. An overview of the CePa algorithm workflow is provided in Figure S5.

References

1. Gu, Z., Liu, J., Cao, K., Zhang, J. & Wang, J. Centrality-Based Pathway Enrichment: a Systematic Approach for Finding Significant Pathways Dominated by Key Genes. *BMC Syst. Biol.* **6**, 1–13 (2012).

2. Sales, G., Calura, E., Cavalieri, D. & Romualdi, C. graphite-A Bioconductor Package to Convert Pathway Topology to Gene Network. *BMC Bioinforma.* **13**, 1–12 (2012).

Acknowledgements

This paper is based on research partially supported by the National Science Foundation under Grant No. NSF-1830676.

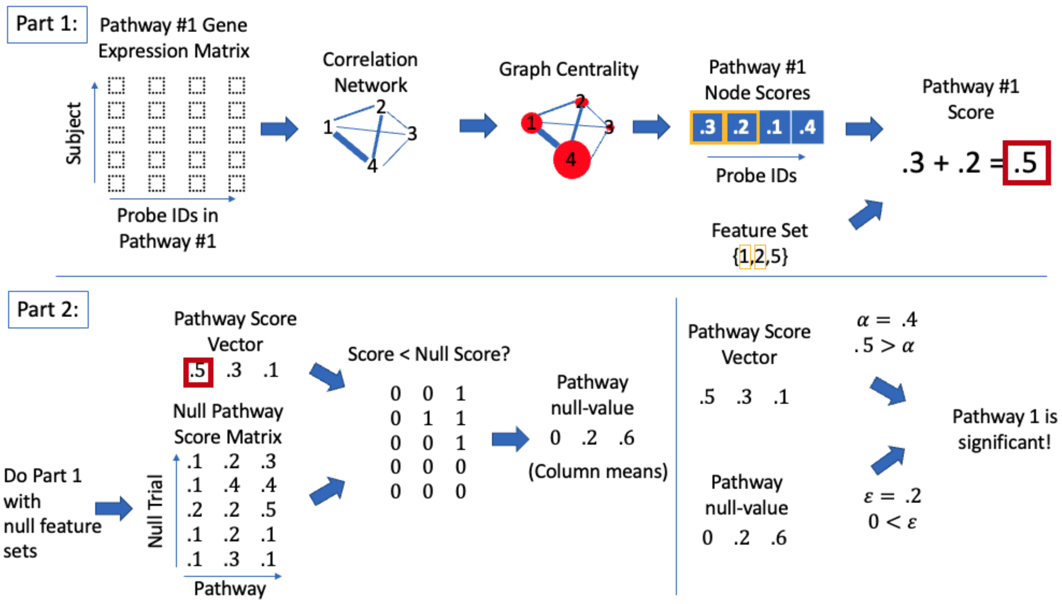

Figure S5. The workflow for CePa.
